# Catalogue of the Graduates of the University of Louisville

**Published:** 1853-04

**Authors:** 


### CATALOGUE OF THE GRADUATES OF THE UNIVERSITY OF LOUISVILLE.

A catalogue of all the graduates of the Medical Department of the University of Louisville, from the origin of the school, in 1837, has been published, and copies may be had by application to the Dean of the Faculty, Dr. L. P. Yandell.

